# *CKLF* as a Prognostic Biomarker and Its Association with Immune Infiltration in Hepatocellular Carcinoma

**DOI:** 10.3390/curroncol30030202

**Published:** 2023-02-22

**Authors:** Dan Li, Shenglan Huang, Chen Luo, Yongkang Xu, Shumin Fu, Kan Liu, Jianbing Wu

**Affiliations:** 1 Department of Oncology, The Second Affiliated Hospital of Nanchang University, Nanchang 330006, China; 2 Jiangxi Key Laboratory of Clinical and Translational Cancer Research, Nanchang 330006, China; 3 Department of General Surgery, The First Affiliated Hospital of Nanchang University, Nanchang 330006, China

**Keywords:** *CMTM* family member, hepatocellular carcinoma, immune infiltration, prognosis, survival

## Abstract

The Chemokine-like factor (*CKLF*)-like MARVEL transmembrane domain-containing (*CMTM*) family, comprising nine members, is involved in the tumorigenesis and progression of various cancers. However, the expression profiles and clinical significance of *CMTM* family members in hepatocellular carcinoma (HCC) are not fully clarified. In this study, the RNA-sequencing and clinical data were downloaded from The Cancer Genome Atlas (TCGA) databases. The Kaplan–Meier method and the Cox proportional hazards regression analysis were used to evaluate the prognostic significance of *CMTM* family members. Single-sample gene set enrichment analysis (ssGSEA) and ESTIMATE algorithms were employed to explore the relationship between *CMTM* family genes and the tumor microenvironment in HCC. Finally, the prognostic *CMTM* family gene expression was further validated by quantitative real-time polymerase chain reaction (qRT-PCR) and immunohistochemical (IHC) staining in clinical HCC tissue specimens. The results indicated that, compared with normal tissues, the expression of *CKLF*, *CMTM1*, *CMTM3*, *CMTM4*, *CMTM7*, and *CMTM8* were significantly upregulated in HCC, while the expression of *CMTM2*, *CMTM5*, and *CMTM6* were significantly downregulated in HCC. Univariate and multivariate Cox regression analysis demonstrated that *CKLF* was an independent prognostic biomarker for the overall survival (OS) of HCC patients. In HCC, the expression of *CKLF* was found to be correlated with immune cell infiltration, immune-related functions, and immune checkpoint genes. The qRT-PCR and IHC confirmed that *CKLF* was highly expressed in HCC. Overall, this research suggested that *CKLF* is involved in immune cell infiltration and may serve as a critical prognostic biomarker, which provides new light on the therapeutics for HCC.

## 1. Introduction

Liver cancer is one of the most common malignancies of the digestive system and the fourth cause of death due to cancer worldwide [1]. Hepatocellular carcinoma (HCC) is the main type of primary liver cancer, accounting for approximately 75–85% of all cases [2]. Hepatectomy, radiofrequency ablation (RFA), liver transplantation, transcatheter arterial chemoembolization (TACE), and targeted therapy are the main treatments for HCC, but the 5-year overall survival rate of HCC patients remains poor [3,4]. In addition, HCC is usually asymptomatic in its early stages, leading to a poor prognosis for most patients who are already in the middle and advanced stages at the time of diagnosis [5]. Therefore, it is urgent to identify robust diagnostic biomarkers and effective therapeutic targets to improve the prognosis and curative effect of HCC patients.

Chemokine-like factor (*CKLF*)-like MARVEL transmembrane domain-containing (*CMTM*) is a novel gene family which consist of *CKLF* and *CMTM1*-8 [6]. The MARVEL domain of the *CMTM* family comprises four transmembrane helices and is involved in vesicle trafficking and membrane linking [7]. The structural features of the encoded proteins of the *CMTM* family are intermediate between the classical chemokines and the transmembrane 4-superfamily (TM4SF) [8]. *CMTM* family members have been associated with tumor cell proliferation, apoptosis, invasion, and migration [9]. Furthermore, angiogenesis and the recruitment of immune cells are also linked to *CMTM* family members [10,11]. Additionally, previous studies have revealed that the expression and prognostic roles of *CMTM* family members are quite different in various tumors [12]. For instance, overexpression of *CKLF* was detected in HCC and was associated with poor survival in HCC patients [13]. Research reported that high expression of *CMTM1* in glioblastoma enhanced aggressive tumor behavior, resulting in a worse prognosis [14]. Increased expression of *CMTM3* in pancreatic carcinoma was correlated with lower pathological grade, higher recurrence/distant metastasis rate, and poorer survival time [15]. Additionally, abnormal expression of *CMTM4* and *CMTM6*, which serve as a regulator of programmed death-ligand 1 (PD-L1), has been reported to be associated with survival and they may be a new target for prognostic biomarkers and immunotherapy [16,17,18]. Similarly, previous studies found that *CKLF* had broad chemotactic activity on many cells, including lymphocytes, macrophages, and neutrophils, and was involved in promoting the proliferation and differentiation of human bone marrow cells [19]. In addition, *CKLF* expression was increased in monocytes and activated CD4+ and CD8+ lymphocytes [20]. These studies suggest that some *CMTM* family genes (*CKLF*, *CMTM4*, and *CMTM6*) might not only predict prognosis, but also be considered as a potential immune target, which indicated its high prospect of clinical application.

In this study, the expression and prognostic value of *CMTM* family members in HCC were comprehensively investigated through public resources and multiple bioinformatics analyses. Furthermore, we discuss the correlation of *CMTM* family member expression with immune cell infiltration, immune-related functions, and immune checkpoint genes in HCC. Finally, we conducted quantitative real-time polymerase chain reaction (qRT-PCR) and immunohistochemistry (IHC) experiments to validate the prognostic *CMTM* family gene expression in HCC. Collectively, the purpose of this experiment was to determine whether the *CMTM* gene could be a new potential prognostic biomarker for HCC and hopefully contribute to the screening of a prognostic indicator that could be used to predict survival and guide immunotherapy in HCC patients.

## 2. Materials and Methods 

### 2.1. Data Sources

This study obtained the RNA-sequence data of 374 HCC tumor tissues and 50 normal tissues from The Cancer Genome Atlas (TCGA) (https://portal.gdc.cancer.gov, accessed on 20 April 2022) [21]. RNA-sequence data in fragments per kilobase million (FPKM) format were converted to transcripts per million (TPM) formats, and then log2 transformed for analysis. Meanwhile, the clinical data were also acquired from the TCGA database, including age, gender, histological grade, tumor (T) stage, node (N) stage, metastasis (M) stage, survival time, survival status, and so on.

### 2.2. Differential Expression Data of CMTM Family Members in HCC

The differential expression of *CMTM* family between HCC tissues and paired normal tissues was identified using the limma package [22] in R 4.0.2 software. *CMTM* family gene expression in tumor and normal tissues was analyzed by Wilcoxon rank sum test. The results were plotted as pairwise boxplot and heatmap, which were visualized by the ggplot2 [23] and pheatmap packages [24].

### 2.3. Clinicopathological Characteristics Analysis of CMTM Family Members in HCC

UALCAN (http://ualcan.path.uab.edu, accessed on 25 April 2022) database was used to investigate the association between the mRNA expression of *CMTM* family members and clinicopathological features (cancer stage and tumor grade) in patients with HCC [25]. Additionally, TCGA database was used to complement the association between the mRNA expression of CMTM family members and clinicopathological characteristics of HCC patients, such as age, gender, AFP, and vascular invasion status. Expression differences were verified by Student’s *t*-test or Wilcoxon signed-rank test, and *p* < 0.05 was considered statistically significant.

### 2.4. Survival and Prognostic Analysis of CMTM Family Members in HCC

The diagnostic receiver operating characteristic (ROC) curves and the time-dependent receiver operating characteristic (ROC) curve were performed using the “pROC” and “timeROC” R packages [26] to evaluate the predictive value of *CMTM* family members expression for diagnosis and prognosis of HCC. An AUC of 0.5–0.7 was indicative of low diagnostic accuracy, an AUC of 0.7–0.9 was indicative of moderate diagnostic accuracy, and an AUC higher than 0.9 was indicative of high diagnostic accuracy.

Kaplan–Meier plotters (http://kmplot.com/analysis/, accessed on 25 April 2022) were used to evaluate the prognostic significance of the expression of *CMTM* family genes regarding OS (overall survival) and progression-free survival (PFS) [27]. Based on the median expression value of *CMTM* family members, HCC patients were divided into high- and low-expression groups and verified by Kaplan–Meier survival curves and log-rank tests. The number-at-risk cases, log-rank *p*-value, and hazard ratio (HR) with 95% confidence intervals (CIs) were presented in every survival curve plotting.

Cox proportional hazards regression analyses was used to assess the potential of *CMTM* family members as independent prognostic factors in patients diagnosed with HCC. First, the relationship between *CMTM* members and clinicopathological parameters (including clinical stage, grade gender, and age) and survival of HCC patients was evaluated using univariate Cox proportional hazards regression analysis. Subsequently, clinical characteristics with a *p*-value < 0.05, including *CMTM* expression, were included for multivariate analysis. Similarly, HCC clinical samples were performed to validate whether *CKLF* was an independent predictive factor for the prognosis of HCC patients by the same method mentioned above. A *p* < 0.05 was considered statistically significant. 

### 2.5. The Investigation of CMTM Family Members with Tumor Microenvironment and Immune Checkpoint Genes in HCC

The GSVA packages [28] in R language was applied to estimate the correlation between the expression of the *CMTM* family members and 24 types of immune cell infiltration. The single-sample gene set enrichment analysis (ssGSEA) algorithm was used to assess 16 types of immune cell infiltration and 13 immune functions between the high and low expressions of *CMTM* family members [29]. Afterwards, we used the ESTIMATE algorithm to calculate the ImmuneScore, StromalScore, and ESTIMATEScore in HCC samples, which analyzes the tumor microenvironment (TME) status of HCC [30]. In addition, we obtained a heatmap presenting the correlation between the immune checkpoint genes and *CMTM* family members [31]. We also compared the expression of immune checkpoint genes between the high and low expressions of *CMTM* family members, and the results were presented as a box chart.

### 2.6. Gene Set Enrichment Analysis (GSEA)

To investigate the molecular mechanisms by which *CMTM* family members were involved in the development and progression of HCC, we explored the signal pathways related to *CMTM* members in HCC via GSEA (version 4.0.1; http://software.broadinstitute.org/gsea/index.jsp, accessed on 1 May 2022) [32]. The c2.cp.kegg.v7.5.symbols.gmt was utilized as the reference gene set. The results with a false discovery rate (FDR) < 0.05 and *p* < 0.05 were considered statistically significant. 

### 2.7. Patients and Tumor Tissues

The HCC tissues and adjacent normal tissues of 41 patients with HCC were collected from the Second Affiliated Hospital of Nanchang University. Patients with HCC confirmed by histopathological diagnosis and without adjuvant anticancer treatment such as TACE, radiotherapy, and chemotherapy prior to surgery were selected for the study. The experiments were approved by the Ethics Committee of the Second Affiliated Hospital of Nanchang University and conducted following the Declaration of Helsinki. All participants signed an informed consent form.

### 2.8. qRT-PCR

HCC tissue samples were extracted for total RNA according to the product manual of TRIzol Reagent (Invitrogen). RNA was inversely transcribed into complementary DNA (cDNA) using PrimeScript™ RT Kit (TaKaRa, Kusatsu, Japan; RR047A). TB Green^®^ Premix Ex Taq™ II kit (TaKaRa, RR820A) was used to conduct qRT-PCR. Additionally, all PCRs were amplified by the CFX96 real-time PCR detection system with the following reaction conditions: an initial step of 94.0 °C for 30 s, followed by 40 cycles of 94.0 °C for 4 s, 58.0 °C for 15 s, and 72 °C for 15 s. The results were analyzed by 2^−ΔΔCt^. The primer sequences of genes are provided in Table 1.

### 2.9. Immunohistochemistry Staining

IHC analysis of *CKLF* procedures was performed as described previously [33]. Antibodies used were as follows: rabbit antibody *CKLF* (1:200 primary antibody dilution, abs138894, Absin, Shanghai, China).

### 2.10. Statistical Analysis

All statistical analyses were performed in R software version 4.0.2 and GraphPad Prism software version 7.0 and SPSS software version 22.0. Paired *t*-test, Wilcoxon rank sum test, or one-way ANOVA was used to analyze the differences between groups. Spearman correlation analysis was applied to evaluated correlations. Survival rates were assessed using Kaplan–Meier (K-M) curves and the log-rank test. Univariate and multivariate analyses were conducted by Cox proportional hazards regression model. Variables with prognostic significance from univariate analysis were incorporate into subsequent multivariate analysis, and a *p* < 0.05 was considered statistically significant.

## 3. Result

### 3.1. The Expression of CMTM Family Members in HCC

**Figure 1:**
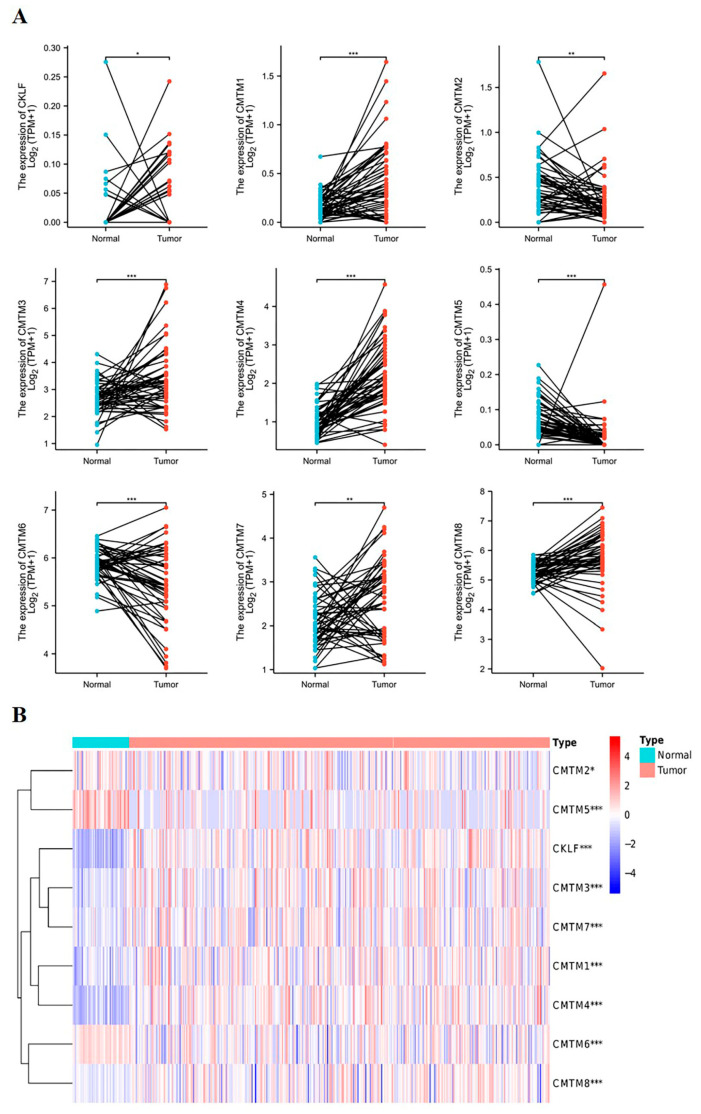
The expression profile of *CMTM* family members in HCC tissues based on TCGA database. The pairwise boxplot shows the mRNA expression of *CMTM* family in 50 paired HCC tissues and normal tissues (**A**). The heatmap shows the expression of *CMTM* family members between HCC tissues and normal tissues (**B**). The darkness of color indicates the expression level of the gene (red indicates high and blue low). * *p* < 0.05, ** *p* < 0.01, *** *p* < 0.001. HCC, hepatocellular carcinoma. *CMTM*, Chemokine-like factor-like MARVEL transmembrane domain-containing family.

First, we used the TCGA database to analyze the expression of *CMTM* family members in paired HCC tissues and normal tissues. The paired data (50 case) results found that the mRNA expression of *CKLF*, *CMTM1*, *CMTM3*, *CMTM4*, *CMTM7*, and *CMTM8* was significantly increased in HCC tissues compared with normal tissues. However, the mRNA expression of *CMTM2*, *CMTM5*, and *CMTM6* was evidently decreased in HCC tissues compared to normal tissues (Figure 1A). The heatmap displayed the differential expression of *CMTM* family members between the HCC tissues and normal tissues (Figure 1B).

### 3.2. Relationship between the mRNA Expression of CMTM Family Members and Clinicopathological Parameters of HCC Patients 

**Figure 2:**
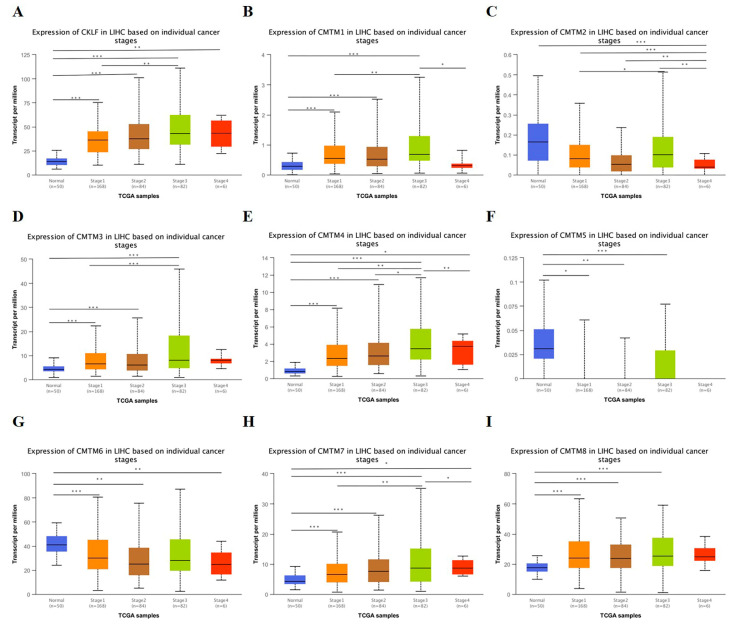
Association of mRNA expression of *CMTM* family members with cancer stage of HCC patients in UALCAN database (**A**–**I**). * *p* < 0.05, ** *p* < 0.01, *** *p* < 0.001. HCC, hepatocellular carcinoma. *CMTM*, Chemokine-like factor-like MARVEL transmembrane domain-containing family.

**Figure 3:**
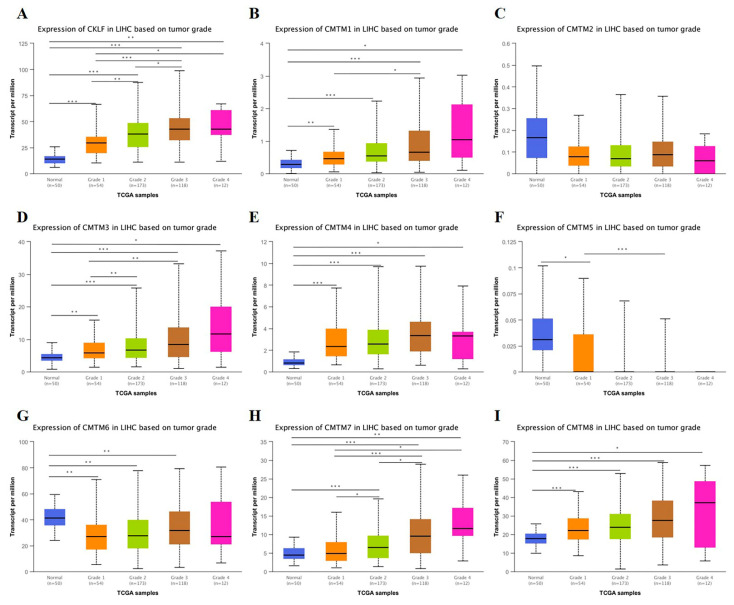
Association of mRNA expression of *CMTM* family members with tumor grade of HCC patients in UALCAN database (**A**–**I**). * *p* < 0.05, ** *p* < 0.01, *** *p* < 0.001. HCC, hepatocellular carcinoma. *CMTM*, Chemokine-like factor-like MARVEL transmembrane domain-containing family.

We analyzed the relationship between the mRNA expression of *CMTM* family members and clinicopathological parameters (cancer stage and tumor grade) of HCC patients based on the UALCAN database. As presented in Figure 2, the mRNA expression of *CKLF*, *CMTM1*, *CMTM3*, *CMTM4*, and *CMTM7* was correlated with the cancer stage, revealing that patients with more advanced cancer stages tended to have higher mRNA expression of the *CMTM* family. As for the relationship between mRNA expression of *CMTM* family members and tumor grade, as shown in Figure 3, the results indicated that mRNA expressions of *CKLF*, *CMTM1*, *CMTM3*, and *CMTM7* was significantly related to tumor grade. As tumor grade increased, the mRNA expression of *CKLF*, *CMTM1*, *CMTM3*, and *CMTM7* tended to be higher. Following that, we used the TCGA database to explore the relationship between the expression of *CMTM* family members and clinicopathological features of HCC, including age, gender, APF, and vascular invasion status. The results showed that the expressions of *CMTM3* and *CMTM7* were significantly lower in HCC patients over 60 years of age (Supplementary Figure S1). We found that the expressions of *CMTM1*, *CMTM3*, and *CMTM4* were significantly higher in the females compared to males (Supplementary Figure S2). Additionally, the patients with a high expression of *CMTM3*, *CMTM4*, *CMTM6*, *CMTM7*, and *CMTM8* were associated with a higher AFP level (>400 ng/mL vs. ≤400 ng/mL) (Supplementary Figure S3). However, the expressions of *CMTM* family members were not associated with vascular invasion status in HCC patients (Supplementary Figure S4).

### 3.3. Diagnostic Capacity of CMTM Family Members in HCC

**Figure 4:**
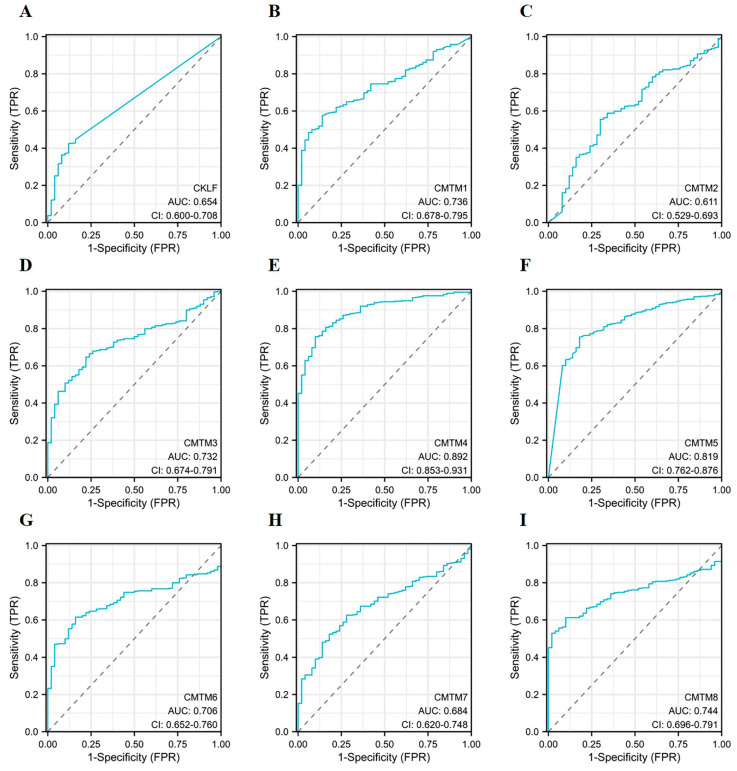
Diagnostic capacity of *CMTM* family members in HCC. ROC curves of *CMTM* family members in HCC (**A**–**I**). ROC, receiver operating characteristic. AUC, area under the curve. TPR, true-positive rate. FPR, false-positive rate. HCC, hepatocellular carcinoma. *CMTM*, Chemokine-like factor-like MARVEL transmembrane domain-containing family.

Receiver operator characteristic (ROC) curves were utilized to assess the diagnostic efficacy of *CMTM* family members in HCC. The area under ROC curve (AUC) was used to illustrate the results of the ROCs. As can be observed in Figure 4, *CMTM4* and *CMTM5* were found to have a high predictive power (AUC > 0.8). Of them, the AUC of *CMTM4* was the highest, with a value of 0.892. Unfortunately, the time-dependent ROC curve analysis revealed that the AUC values for the predicted 1-year OS and PFI of HCC patients based on the expression of *CMTM* family members has a low diagnostic accuracy, with an AUC < 0.7 (Supplementary Figure S5).

### 3.4. Prognostic Value of CMTM Family Members in HCC

**Figure 5:**
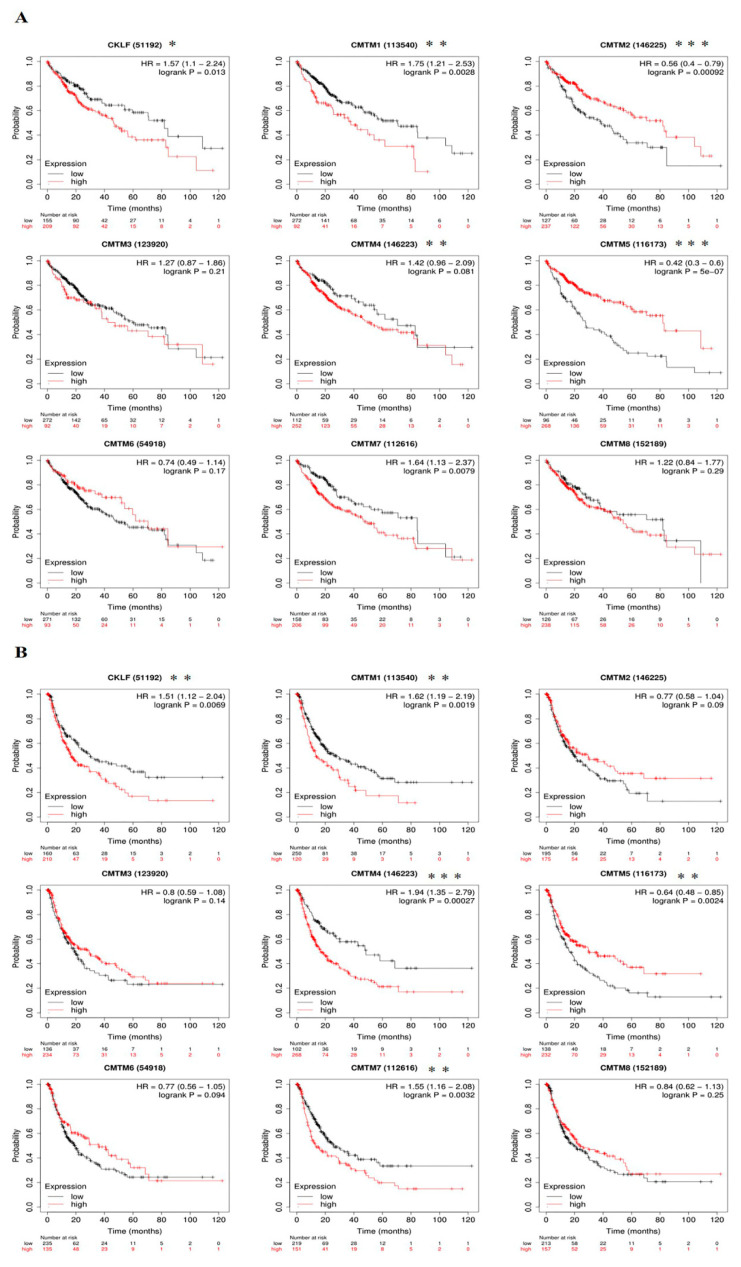
Prognostic value of *CMTM* family members in HCC patients. Kaplan–Meier survival curves of OS (**A**) and PFS (**B**) for the expression of *CMTM* family members in patients with HCC. * *p* < 0.05, ** *p* < 0.01, *** *p* < 0.001. HCC, hepatocellular carcinoma. OS, overall survival. PFS, progression-free survival. *CMTM*, Chemokine-like factor-like MARVEL transmembrane domain-containing family.

We first used the Kaplan–Meier plotter online database to analyze the associations between *CMTM* family expressions and patients’ survival. The result from the Kaplan–Meier survival curves revealed that high mRNA expressions of *CKLF* (*p* = 0.013), *CMTM1* (*p* = 0.0028), and *CMTM7* (*p* = 0.0079) were significantly associated with a worse OS in patients with HCC, while high mRNA expressions of *CMTM2* (*p* < 0.001) and *CMTM5* (*p* < 0.001) were remarkably associated with a better OS (Figure 5A). Furthermore, patients with high expressions of *CKLF* (*p* = 0.0069), *CMTM1* (*p* = 0.0019), *CMTM4* (*p* < 0.001), and *CMTM7* (*p* = 0.0032) were correlated with shorter PFS. However, high expression of *CMTM5* (*p* = 0.0024) in HCC patients was correlated with longer PFS (Figure 5B).

To investigate whether *CMTM* family members had independent predictive power for the prognosis of HCC patients, we included *CMTM* family gene expression and clinical information of HCC patients, including age, gender, tumor grade, and cancer stage in univariate and multivariate Cox analyses. Univariate Cox analysis revealed that the expression of *CMTM1*, *CMTM4*, *CMTM7*, and cancer stage were significantly associated with poor prognosis in patients with HCC (*p* < 0.05). However, multivariate Cox regression analysis showed that *CMTM* family members were not an independent prognostic factor for predicting PFI (*p* > 0.05, Supplementary Table S1). Next, Univariate Cox analysis revealed that *CKLF*, *CMTM7*, and pathological stage were risk factors for OS in patients with HCC (*p* < 0.05). Multivariate Cox regression analysis verified that *CKLF* was an independent risk factor that affected the OS in HCC patients (Table 2). Thus, we focused on *CKLF* for our subsequent analysis.

### 3.5. Association of CKLF Expression with Tumor Microenvironment in HCC

**Figure 6:**
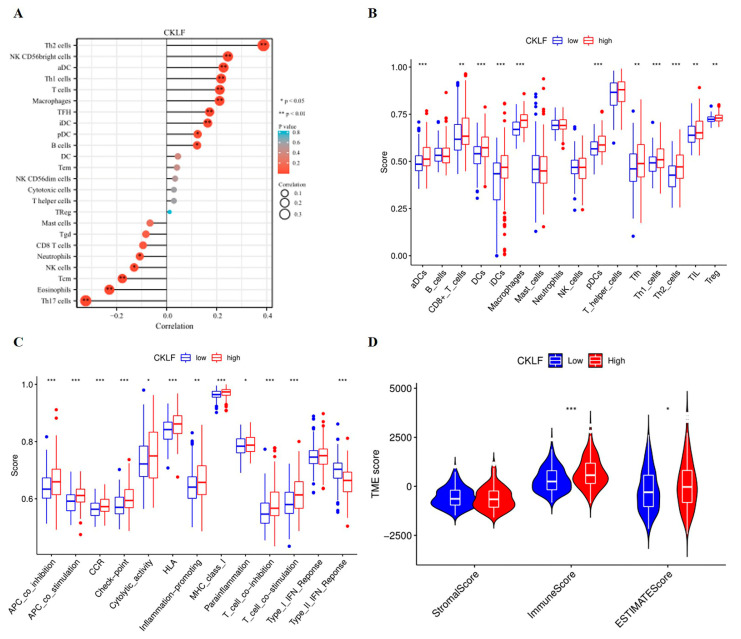
Association between the expression of *CKLF* and the tumor microenvironment in HCC. The lollipop chart shows the correlation between *CKLF* expression and immune cell infiltration (**A**). The expression of immune cells infiltration in high- and low-*CKLF*-expression groups (**B**). Immune-related functional analyses between high- and low-*CKLF*-expression groups (**C**). ESTIMATE algorithm was performed to analyze the association between the two groups in Immune scores, Stromal scores, and ESTIMATE scores (**D**). * *p* < 0.05, ** *p* < 0.01, *** *p* < 0.001. HCC, hepatocellular carcinoma. *CMTM*, Chemokine-like factor-like MARVEL transmembrane domain-containing family.

To explore the effects of the *CKLF* expression in the tumor microenvironment, we first analyzed the correlation between the expression of *CKLF* and immune cell infiltration. We found that *CKLF* expression correlated with most immune cells (Figure 6A). Then, we used the ssGSEA algorithm to analyze the expression levels of immune infiltrating cells in different *CKLF* expression groups. The results found that the high-*CKLF*-expression group had higher levels of infiltration of aDCs, DCs, pDCs, iDCs, CD8+T cells, Macrophages, Tfh, TIL, Th1_cells, Th2_cells, and Treg cells than the low-*CKLF*-expression group (Figure 6B). As can be observed in Figure 6C, compared with the low-*CKLF*-expression group, the high-*CKLF*-expression group revealed higher scores in multiple immune functions, such as checkpoint and Cytolytic activity. In contrast, type II IFN response showed higher activity in the low-*CKLF*-expression group than in the high-*CKLF*-expression group. Additionally, ESTIMATE analysis demonstrated that the Immune and ESTIMATE scores in the high-*CKLF*-expression group were higher than those in the low-*CKLF*-expression group (Figure 6D).

### 3.6. Correlation Analysis of CKLF Expression with Immune Checkpoint Genes

**Figure 7:**
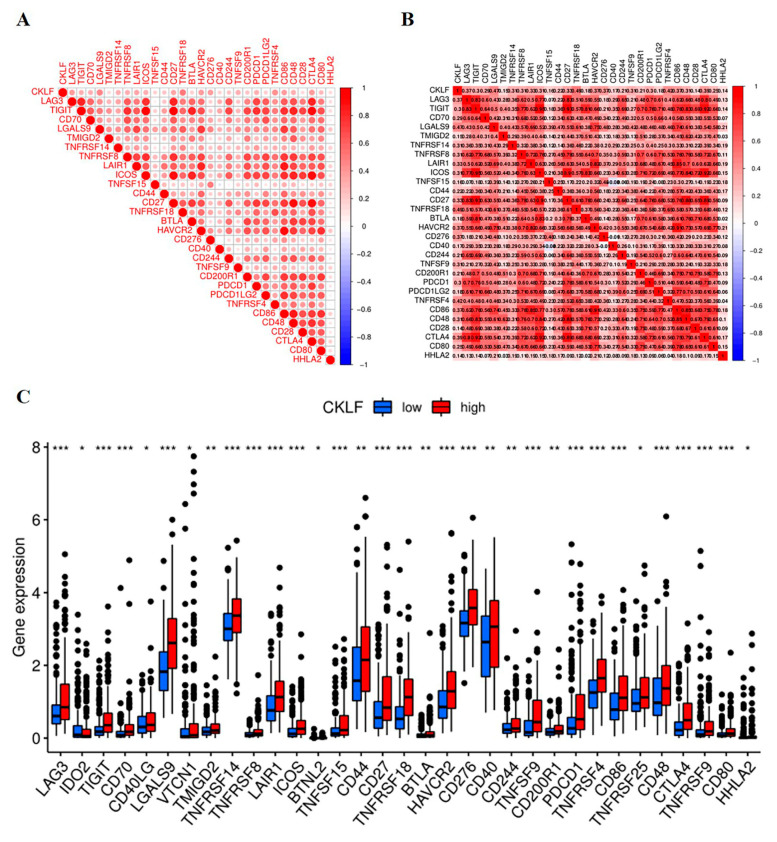
Relationship between the expression of *CKLF* and immune checkpoint genes in HCC. The correlation of *CKLF* expression with immune checkpoint genes (**A**,**B**). The expression of immune checkpoint genes in different *CKLF* expression groups (**C**). * *p* < 0.05, ** *p* < 0.01, *** *p* < 0.001. HCC, hepatocellular carcinoma. *CMTM*, Chemokine-like factor-like MARVEL transmembrane domain-containing family.

We next performed a correlation analysis of *CKLF* expression and immune checkpoint molecules. Our results demonstrated that *CKLF* was positively associated with some immune checkpoint molecules, including CTLA4, HAVCR2, LAG3, PDCD1, and CD276 (all *p*-values < 0.05 Figure 7A,B). In addition, we also compared the expression of immune checkpoint molecules between the high- and low-*CKLF*-expression groups. The results revealed that the expression level of most immune checkpoint genes in the high-*CKLF*-expression group was higher than that in the low-*CKLF*-expression group (Figure 7C).

### 3.7. Exploration of Molecular Mechanisms of CKLF in HCC

**Figure 8:**
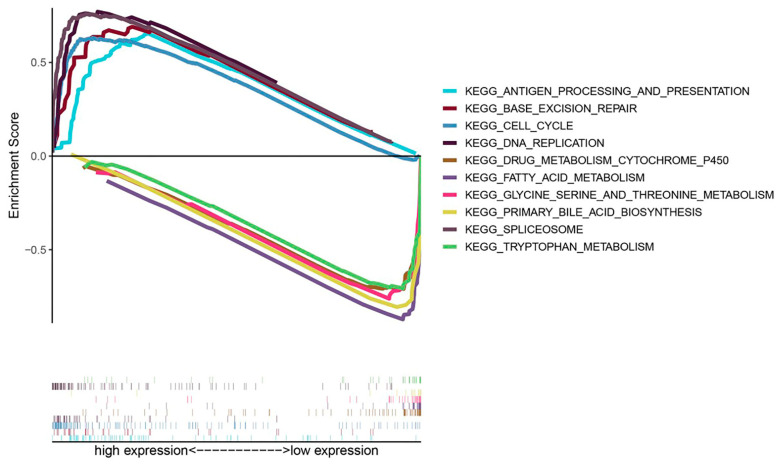
Enrichment maps from KEGG pathways associated with *CKLF* based on GSEA, including enrichment scores and gene sets. KEGG, Kyoto Encyclopedia of Genes and Genomes. GSEA, gene set enrichment analysis.

To clarify the possible molecular mechanisms of *CKLF* expression affecting the tumorigenesis of HCC, we performed a GSEA analysis. The results revealed that a high expression of *CKLF* was positively associated with antigen processing and presentation, base excision repair, cell cycle, DNA replication and spliceosome, and negatively related to metabolism-related pathways, including drug metabolism cytochrome P450, fatty acid metabolism, glycine serine and threonine metabolism, primary bile acid biosynthesis, and tryptophan metabolism (Figure 8).

### 3.8. Experiment Validation of the mRNA Expression and Protein Levels of CKLF in HCC

**Figure 9:**
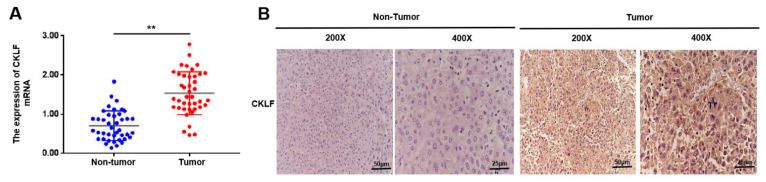
*CKLF* is overexpression in human HCC tissues. *CKLF* mRNA expression was analyzed by qRT-PCR assay in HCC tissues and the corresponding normal tissues of 41 patients with HCC (**A**). IHC method was used to detect the expression of *CKLF* in cancer and the corresponding normal tissues of 41 patients with HCC (**B**). ** *p* < 0.01. HCC, hepatocellular carcinoma. qRT-PCR, quantitative real-time polymerase chain reaction. IHC, immunohistochemical.

To further confirm the bioinformatics analysis results and determine the correlation between *CKLF* expression and HCC, we first compared the expression of *CKLF* in cancer tissues and adjacent tissues taken from 41 HCC patients. The qRT-PCR revealed that the mRNA expression level of *CKLF* was elevated in HCC tissue samples compared with the corresponding adjacent tissues (Figure 9A). Furthermore, immunohistochemistry (IHC) results revealed that *CKLF* was highly expressed in HCC tissues compared to the adjacent nontumor tissues and was mainly localized in the cytosol (Figure 9B). Then, we explored the relationship between the *CKLF* expression and clinicopathological parameters. It was found that there were significant differences in TNM stage between the high- and low-*CKLF*-expression groups (*p* = 0.024, Supplementary Table S2). Kaplan–Meier survival analysis showed that survival rate of patients with high *CKLF* expression was worse than that of those with lower *CKLF* expression (*p* < 0.001, Supplementary Figure S6). Univariate and multivariate Cox regression analyses were performed to investigate whether *CKLF* was an independent predictive factor for the prognosis of HCC patients. The results of the adjustment for conventional clinical patterns, including gender, diagnostic age, pathologic stage, vascular invasion, and serum AFP level, HBV infection, Child–Pugh score, tumor number, and liver cirrhosis status. Univariate analysis results showed that the *CKLF* and TNM stage were associated with poor outcome in HCC patients. Subsequently, clinical characteristics with a *p*-value < 0.05 were included for multivariate analysis, and the results showed that *CKLF* and TNM stage acted as an independent prognostic factor of poor prognosis for HCC patients (Supplementary Table S3). In conclusion, the findings demonstrated that *CKLF* expression is upregulated in HCC and is implicated in the progression of HCC.

## 4. Discussion

With the rapid development of high-throughput sequencing technology, people have reached a new level of understanding of genes or genomes [34]. Exploring the molecular features of disease and individual genetic composition plays an important role in determining prognostic indicators and therapeutic targets for various malignancies [35]. Thus, our research focused on revealing the expression pattern and prognostic value of *CMTM* family genes in HCC through bioinformatics analysis, which is important to identifying prognostic biomarkers and therapeutic targets for HCC patients.

The *CMTM* family genes play a crucial role in various physiological and pathological processes [36]. Recently, aberrant expression of *CMTM* genes has been demonstrated in a variety of tumors and has been implicated in tumorigenesis, progression, metastasis, and prognosis [9]. For instance, previous studies have found that *CKLF* was upregulated in HCC tissues and was associated with tumor stage and patient survival [13]. *CMTM1* was upregulated in glioblastoma and *CMTM1* overexpression enhanced aggressive tumor behavior, which was associated with worse overall survival in glioblastoma patients [14]. Other studies have revealed that *CMTM2* was downregulated in HCC tissues compared to control tissues, and its expression was associated with pathological grades in HCC patients [37]. Some studies have demonstrated that the overexpression of *CMTM3* in pancreatic carcinoma was correlated with high recurrence, distant metastasis rate, low pathological grade, and poor survival times [15]. *CMTM4* was upregulated in HCC; the higher the expression was, the poorer patient survival was associated. The related function may be that *CMTM4* effectively escapes the T-cell-mediated cytotoxicity by stabilizing the expression of PD-L1. Hence, *CMTM4* expression could be instructive for future anti-PD-L1 immunotherapy [17]. *CMTM5*, which was lowly expressed in HCC, can inhibit the proliferation, invasion, and migration of HepG2 cells in vitro and was suppressed by the upregulated microRNA-10b-3p [38]. Additionally, *CMTM6* knockdown could significantly reduce PD-L1 expression and increase infiltration of CD8+ and CD4+ T-cells, thereby enhancing the antitumor immunity in HNSCC [39]. *CMTM7* participated in EGFR-AKT signaling in nonsmall-cell lung cancer; knocking down *CMTM7* could reduce Rab5 activation, which promoted tumor growth and metastasis [40]. *CMTM8* was found to be downregulated in gastric cancer tissues rather than that in nontumor tissues, and the expression of *CMTM8* was associated with metastasis of gastric cancer and prognosis of GC patients [41]. In our study, we found that all the *CMTM* genes were highly expressed in HCC except *CMTM2*, *CMTM5*, and *CMTM6* according to data from the TCGA database. The expression of *CKLF*, *CMTM1*, *CMTM3*, and *CMTM7* was correlated with cancer stage and tumor grade. Kaplan–Meier curves showed that the overexpression of *CKLF*, *CMTM1*, and *CMTM7* predicted poor OS and PFS in patients with HCC. Further, univariate and multivariate Cox regression analyses confirmed that *CKLF* was an independent factor for OS in HCC. Subsequently, we confirmed that the expression of *CKLF* was upregulated in HCC clinical tissues by RT-qPCR and IHC. Clinical data further confirmed that the high expression of *CKLF* was associated with poor prognosis. Multivariate Cox analysis also indicated that *CKLF* as an independent prognostic factor which can predict prognosis in HCC patients. Therefore, these results suggest that *CKLF* could be used as a predictive biomarker of the diagnosis and prognosis of HCC patients.

Tumor-immune-infiltrating cells are an important component of the tumor microenvironment (TME) and have been shown to be associated with tumor development and prognosis [42]. Our results revealed that *CKLF* expression was negatively correlated with the levels of neutrophils and NK cells. Neutrophils play an important antitumor role by activating an immune response against tumor cells and directly lysing tumor cells [43]. NK cells are an essential antitumor immune cell that primarily mediates immune surveillance of malignancies [44]. In addition, our data also demonstrated that *CKLF* expression was positively correlated with dendritic cells, tumor-associated macrophages (TAMs), and Th2 and Th1 cells. Dendritic cells were the most effective antigen-presenting cells and initiated antitumor immunity by activating CD8 T cells [45]. In the TME, tumor-associated macrophages (TAMs) could be observed at all stages of liver cancer progression and may mediate immune escape, indicating a key role for TAMs during tumor progression of HCC [46]. Previous studies have shown that *CKLF* had broad spectrum of chemotactic activity for many cells, including lymphocytes, macrophages, and neutrophils [47]. *CKLF* had been reported to activate neutrophils through the mitogen-activated protein kinase (MAPK) pathway and was highly expressed in activated CD8+, CD4+ T cells, and monocyte [19]. Additionally, *CKLF* plays a crucial role in the maturation of DCs as well as in the activation of T-lymphocytes and is involved in humoral immune response and the formation of germinal centers by acting on GC-Th cells [8,48]. Previous studies were consistent with the results of immune infiltration analysis in our study, suggesting that *CKLF* is closely associated with immune regulation, which may contribute to promote tumor proliferation and metastasis of HCC.

A growing body of research shows that immune checkpoint inhibitors (ICIs) have made great progress in the treatment of various cancers, including HCC [49]. ICIs, such as PD-1, PD-L1, and CTLA-4 antibodies, have been shown to promote the activity of potent T-cells and inhibit immunosuppression in the tumor microenvironment [50]. In the IMBRAVE150 trial, HCC patients treated with the combination of atezolizumab (PD-L1 inhibitor) with bevacizumab (VEGF inhibitor) had a favorable prognosis compared to patients treated with sorafenib alone [51]. In spite of these significant advances, only a minority of HCC patients benefit from ICIs therapy [52,53]. Additionally, there are no credible biomarkers for predicting immunotherapy response to guide personalized therapy and improve clinical outcome [54]. Based on the exploratory endpoints of HCC trials, several potential biomarkers have been proposed, such as PD-L1 expression and specific genomic alterations [55,56]. The combined PD-L1 positive score positive score of PD-L1 could predict the response to pembrolizumab and was associated with PFS of HCC patients [57]. In this study, the expression of *CKLF* was significantly correlated with some immune-related genes, which is of great significance. These findings suggest that *CKLF* could be used as a therapeutic target to predict the therapeutic efficacy of ICIs for HCC.

Our study sheds light on the potential role of *CKLF* in tumor immunology and its potential to serve as a tumor biomarker and a new therapy target of HCC. However, there are limitations in this study. Firstly, this study was primarily based on data from several public databases, and confounder factors might lead to biases. Secondly, although the association between the expression of *CKLF* and immune cell infiltration was investigated using bioinformatics, the molecular mechanisms and biological function needs to be verified by in vivo and in vitro experiments. Finally, we also need to perform stratified analysis, which is crucial for the need for more elaborate and individualized methods in well-designed clinical trials.

## 5. Conclusions

Taken together, we comprehensively analyzed the expression levels and prognostic values of *CMTM* family members in HCC. We identified that *CKLF* is an important diagnostic and independent prognostic biomarker in HCC patients. Additionally, we also revealed that *CKLF* expression was associated with immune cell infiltration and immune checkpoint genes in HCC. Our research provided new insight into the clinical application of *CKLF* as a prognosis biomarker and therapeutic target in patients with HCC.

## Figures and Tables

**Table 1:** Sequences of qRT-PCR primers.

Gene Name	Sequence (5′-3′)
*CKLF*	F: CAGCAGTATGCTGTCTTGCCGA; R: TTTTTCATGCACAGGCTTTTTCTGG
GADPH	F: GGAGCGAGATCCCTCCAAAAT; R: GGCTGTTGTCATACTTCTCATGG

qRT-PCR, quantitative real-time reverse transcription polymerase chain reaction. F, forward primer. R, reverse primer.

**Table 2:** Univariate and Multivariate Cox proportional hazards regression analysis of *CMTM* family members and clinical characteristics for overall survival in HCC.

Characteristics	Univariate Analysis		Multivariate Analysis	
	Hazard Ratio (95% CI)	*p* Value	Hazard Ratio (95% CI)	*p* Value
Age				
≤60	Reference	-	-	-
>60	1.286 (0.876–1.887)	0.199	-	-
Gender				
Female	Reference	-	-	-
Male	0.816 (0.552–1.206)	0.307	-	-
Histologic grade				
G1	Reference	-	-	-
G2	1.229 (0.668–2.261)	0.507	-	-
G3	1.234 (0.656–2.320)	0.515	-	-
G4	1.767 (0.625–4.992)	0.283	-	-
Pathologic stage				
Stage I	Reference	-	Reference	-
Stage II	1.511 (0.905–2.523)	0.114	1.482 (0.887–2.477)	0.133
Stage III	2.708 (1.742–4.211)	**<0.001**	2.734 (1.756–4.255)	**<0.001**
Stage IV	5.612 (1.722–18.292)	**0.004**	4.886 (1.495–15.963)	**0.009**
CKLF	1.985 (1.348–2.922)	**0.001**	1.879 (1.237–2.855)	**0.003**
CMTM1	1.047 (0.716–1.530)	0.813	-	-
CMTM2	1.156 (0.789–1.697)	0.458	-	-
CMTM3	1.053 (0.720–1.539)	0.791	-	-
CMTM4	1.258 (0.859–1.842)	0.238	-	-
CMTM5	1.124 (0.767–1.648)	0.549	-	-
CMTM6	1.373 (0.936–2.015)	0.105	-	-
CMTM7	1.659 (1.164–2.363)	**0.005**	1.289 (0.876–1.898)	0.198
CMTM8	1.060 (0.725–1.550)	0.765	-	-

Bold values stand for *p* < 0.05. HR, hazard ratio. CI, confidence interval. HCC, hepatocellular carcinoma. CMTM, Chemokine-like factor-like MARVEL transmembrane domain-containing family.

## Data Availability

The data of this manuscript can be downloaded from The Cancer Genome Atlas database (https://portal.gdc.cancer.gov/, accessed on 20 April 2022), UALCAN (http://ualcan.path.uab.edu, accessed on 25 April 2022) and Kaplan–Meier plotter (http://kmplot.com/analysis/, accessed on 25 April 2022).

## Supplementary Materials

The following supporting information can be downloaded at: https://www.mdpi.com/article/10.3390/curroncol30030202/s1, Figure S1: Correlation of mRNA expression of CMTM family members with age of HCC patients in TCGA database. * *p* < 0.05. HCC, hepatocellular carcinoma. CMTM, Chemokine-like factor-like MARVEL transmembrane domain-containing family; Figure S2: Correlation of mRNA expression of CMTM family members with gender of HCC patients in TCGA database. ** *p* < 0.01. HCC, hepatocellular carcinoma. CMTM, Chemokine-like factor-like MARVEL transmembrane domain-containing family; Figure S3: Correlation of mRNA expression of CMTM family members with AFP of HCC patients in TCGA database. ** *p* < 0.01, *** *p* < 0.001. HCC, hepatocellular carcinoma. CMTM, Chemokine-like factor-like MARVEL transmembrane domain-containing family; Figure S4: Correlation of mRNA expression of CMTM family members with vascular invasion status of HCC patients in TCGA database. HCC, hepatocellular carcinoma. CMTM, Chemokine-like factor-like MARVEL transmembrane domain-containing family; Figure S5: Time-dependent survival ROC curves to predict 1-year OS (A) and PFI (B) of HCC patients based on based on the expression levels of CMTM family members. OS, overall survival. PFI, progression-free interval (PFI). ROC, receiver operating characteristic. HCC, hepatocellular carcinoma. TPR, true-positive rate. FPR, false-positive rate. CMTM, Chemokine-like factor-like MARVEL transmembrane domain-containing family; Figure S6: Kaplan–Meier survival curves indicated that high expression of CKLF was related to poor prognosis of HCC patients. HCC, hepatocellular carcinoma. Table S1: Univariate and multivariate Cox proportional hazards regression analysis of CMTM family members and clinical characteristics for progression-free interval in HCC; Table S2: The relationship between CKLF expression and clinicopathological characteristics in HCC patients; Table S3: Univariate and multivariate analyses of overall survival in HCC patients (Cox proportional hazards regression model).

## References

[1] Sung H., Ferlay J., Siegel R.L., Laversanne M., Soerjomataram I., Jemal A., Bray F. (2021). Global Cancer Statistics 2020: GLOBOCAN Estimates of Incidence and Mortality Worldwide for 36 Cancers in 185 Countries. CA Cancer J. Clin..

[2] Llovet J.M., Kelley R.K., Villanueva A., Singal A.G., Pikarsky E., Roayaie S., Lencioni R., Koike K., Zucman-Rossi J., Finn R.S. (2021). Hepatocellular carcinoma. Nat. Rev. Dis. Primers.

[3] Craig A.J., von Felden J., Garcia-Lezana T., Sarcognato S., Villanueva A. (2020). Tumour evolution in hepatocellular carcinoma. Nat. Rev. Gastroenterol. Hepatol..

[4] Kulik L., El-Serag H.B. (2019). Epidemiology and Management of Hepatocellular Carcinoma. Gastroenterology.

[5] Li J., Han X., Yu X., Xu Z., Yang G., Liu B., Xiu P. (2018). Clinical applications of liquid biopsy as prognostic and predictive biomarkers in hepatocellular carcinoma: Circulating tumor cells and circulating tumor DNA. J. Exp. Clin. Cancer Res..

[6] Han W., Ding P., Xu M., Wang L., Rui M., Shi S., Liu Y., Zheng Y., Chen Y., Yang T. (2003). Identification of eight genes encoding chemokine-like factor superfamily members 1–8 (*CKLF*SF1-8) by in silico cloning and experimental validation. Genomics.

[7] Sánchez-Pulido L., Martín-Belmonte F., Valencia A., Alonso M.A. (2002). MARVEL: A conserved domain involved in membrane apposition events. Trends Biochem. Sci..

[8] Duan H.J., Li X.Y., Liu C., Deng X.L. (2020). Chemokine-like factor-like MARVEL transmembrane domain-containing family in autoimmune diseases. Chin. Med. J..

[9] Wu J., Li L., Wu S., Xu B. (2020). CMTM family proteins 1-8: Roles in cancer biological processes and potential clinical value. Cancer Biol. Med..

[10] Chrifi I., Louzao-Martinez L., Brandt M.M., van Dijk C., Bürgisser P.E., Zhu C., Kros J.M., Verhaar M.C., Duncker D.J., Cheng C. (2019). *CMTM4* regulates angiogenesis by promoting cell surface recycling of VE-cadherin to endothelial adherens junctions. Angiogenesis.

[11] Burr M.L., Sparbier C.E., Chan Y.C., Williamson J.C., Woods K., Beavis P.A., Lam E., Henderson M.A., Bell C.C., Stolzenburg S. (2017). *CMTM6* maintains the expression of PD-L1 and regulates anti-tumour immunity. Nature.

[12] Li J., Wang X., Wang X., Liu Y., Zheng N., Xu P., Zhang X., Xue L. (2022). CMTM Family and Gastrointestinal Tract Cancers: A Comprehensive Review. Cancer Manag. Res..

[13] Liu Y., Liu L., Zhou Y., Zhou P., Yan Q., Chen X., Ding S., Zhu F. (2019). *CKLF*1 Enhances Inflammation-Mediated Carcinogenesis and Prevents Doxorubicin-Induced Apoptosis via IL6/STAT3 Signaling in HCC. Clin. Cancer Res..

[14] Delic S., Thuy A., Schulze M., Proescholdt M.A., Dietrich P., Bosserhoff A.K., Riemenschneider M.J. (2015). Systematic investigation of CMTM family genes suggests relevance to glioblastoma pathogenesis and *CMTM1* and *CMTM3* as priority targets. Genes Chromosomes Cancer.

[15] Zhou Z., Ma Z., Li Z., Zhuang H., Liu C., Gong Y., Huang S., Zhang C., Hou B. (2021). *CMTM3* Overexpression Predicts Poor Survival and Promotes Proliferation and Migration in Pancreatic Cancer. J. Cancer.

[16] Mezzadra R., Sun C., Jae L.T., Gomez-Eerland R., de Vries E., Wu W., Logtenberg M., Slagter M., Rozeman E.A., Hofland I. (2017). Identification of *CMTM6* and *CMTM4* as PD-L1 protein regulators. Nature.

[17] Chui N.N., Cheu J.W., Yuen V.W., Chiu D.K., Goh C.C., Lee D., Zhang M.S., Ng I.O., Wong C.C. (2022). Inhibition of *CMTM4* Sensitizes Cholangiocarcinoma and Hepatocellular Carcinoma to T Cell-Mediated Antitumor Immunity through PD-L1. Hepatol. Commun..

[18] Peng Q.H., Wang C.H., Chen H.M., Zhang R.X., Pan Z.Z., Lu Z.H., Wang G.Y., Yue X., Huang W., Liu R.Y. (2021). *CMTM6* and PD-L1 coexpression is associated with an active immune microenvironment and a favorable prognosis in colorectal cancer. J. Immunother. Cancer.

[19] Liu D.D., Song X.Y., Yang P.F., Ai Q.D., Wang Y.Y., Feng X.Y., He X., Chen N.H. (2018). Progress in pharmacological research of chemokine like factor 1 (*CKLF*1). Cytokine.

[20] Cai X., Deng J., Ming Q., Cai H., Chen Z. (2020). Chemokine-like factor 1: A promising therapeutic target in human diseases. Exp. Biol. Med..

[21] Tomczak K., Czerwińska P., Wiznerowicz M. (2015). The Cancer Genome Atlas (TCGA): An immeasurable source of knowledge. Contemp. Oncol..

[22] Ritchie M.E., Phipson B., Wu D., Hu Y., Law C.W., Shi W., Smyth G.K. (2015). limma powers differential expression analyses for RNA-sequencing and microarray studies. Nucleic. Acids Res..

[23] Ito K., Murphy D. (2013). Application of ggplot2 to Pharmacometric Graphics. CPT Pharmacomet. Syst. Pharmacol..

[24] Gu Z., Hübschmann D. (2022). Make Interactive Complex Heatmaps in R. Bioinformatics.

[25] Chandrashekar D.S., Bashel B., Balasubramanya S., Creighton C.J., Ponce-Rodriguez I., Chakravarthi B., Varambally S. (2017). UALCAN: A Portal for Facilitating Tumor Subgroup Gene Expression and Survival Analyses. Neoplasia.

[26] Robin X., Turck N., Hainard A., Tiberti N., Lisacek F., Sanchez J.C., Müller M. (2011). pROC: An open-source package for R and S+ to analyze and compare ROC curves. BMC Bioinform..

[27] Lánczky A., Győrffy B. (2021). Web-Based Survival Analysis Tool Tailored for Medical Research (KMplot): Development and Implementation. J. Med. Internet Res..

[28] Hänzelmann S., Castelo R., Guinney J. (2013). GSVA: Gene set variation analysis for microarray and RNA-seq data. BMC Bioinform..

[29] Yi M., Nissley D.V., McCormick F., Stephens R.M. (2020). ssGSEA score-based Ras dependency indexes derived from gene expression data reveal potential Ras addiction mechanisms with possible clinical implications. Sci. Rep..

[30] Yoshihara K., Shahmoradgoli M., Martínez E., Vegesna R., Kim H., Torres-Garcia W., Treviño V., Shen H., Laird P.W., Levine D.A. (2013). Inferring tumour purity and stromal and immune cell admixture from expression data. Nat. Commun..

[31] Jin Z., Sun D., Song M., Zhu W., Liu H., Wang J., Shi G. (2022). Comprehensive Analysis of HOX Family Members as Novel Diagnostic and Prognostic Markers for Hepatocellular Carcinoma. J. Oncol..

[32] Subramanian A., Tamayo P., Mootha V.K., Mukherjee S., Ebert B.L., Gillette M.A., Paulovich A., Pomeroy S.L., Golub T.R., Lander E.S. (2005). Gene set enrichment analysis: A knowledge-based approach for interpreting genome-wide expression profiles. Proc. Natl. Acad. Sci. USA.

[33] Li Q., Chen L., Luo C., Chen Y., Ge J., Zhu Z., Wang K., Yu X., Lei J., Liu T. (2020). TAB3 upregulates PIM1 expression by directly activating the TAK1-STAT3 complex to promote colorectal cancer growth. Exp. Cell Res..

[34] Hong M., Tao S., Zhang L., Diao L.T., Huang X., Huang S., Xie S.J., Xiao Z.D., Zhang H. (2020). RNA sequencing: New technologies and applications in cancer research. J. Hematol. Oncol..

[35] Pleasance E., Bohm A., Williamson L.M., Nelson J., Shen Y., Bonakdar M., Titmuss E., Csizmok V., Wee K., Hosseinzadeh S. (2022). Whole-genome and transcriptome analysis enhances precision cancer treatment options. Ann. Oncol..

[36] Li M., Luo F., Tian X., Yin S., Zhou L., Zheng S. (2020). Chemokine-Like Factor-Like MARVEL Transmembrane Domain-Containing Family in Hepatocellular Carcinoma: Latest Advances. Front. Oncol..

[37] Zhang S., Tian R., Bei C., Zhang H., Kong J., Zheng C., Song X., Li D., Tan H., Zhu X. (2020). Down-Regulated *CMTM2* Promotes Epithelial-Mesenchymal Transition in Hepatocellular Carcinoma. OncoTargets Ther..

[38] Guan L., Ji D., Liang N., Li S., Sun B. (2018). Up-regulation of miR-10b-3p promotes the progression of hepatocellular carcinoma cells via targeting *CMTM5*. J. Cell Mol. Med..

[39] Chen L., Yang Q.C., Li Y.C., Yang L.L., Liu J.F., Li H., Xiao Y., Bu L.L., Zhang W.F., Sun Z.J. (2020). Targeting *CMTM6* Suppresses Stem Cell-Like Properties and Enhances Antitumor Immunity in Head and Neck Squamous Cell Carcinoma. Cancer Immunol. Res..

[40] Liu B., Su Y., Li T., Yuan W., Mo X., Li H., He Q., Ma D., Han W. (2015). *CMTM7* knockdown increases tumorigenicity of human non-small cell lung cancer cells and EGFR-AKT signaling by reducing Rab5 activation. Oncotarget.

[41] Yan M., Zhu X., Qiao H., Zhang H., Xie W., Cai J. (2021). Downregulated *CMTM8* Correlates with Poor Prognosis in Gastric Cancer Patients. DNA Cell Biol..

[42] Hao X., Sun G., Zhang Y., Kong X., Rong D., Song J., Tang W., Wang X. (2021). Targeting Immune Cells in the Tumor Microenvironment of HCC: New Opportunities and Challenges. Front. Cell Dev. Biol..

[43] Que H., Fu Q., Lan T., Tian X., Wei X. (2022). Tumor-associated neutrophils and neutrophil-targeted cancer therapies. Biochim. Biophys. Acta Rev. Cancer.

[44] Liu S., Galat V., Galat Y., Lee Y., Wainwright D., Wu J. (2021). NK cell-based cancer immunotherapy: From basic biology to clinical development. J. Hematol. Oncol..

[45] Fu C., Jiang A. (2018). Dendritic Cells and CD8 T Cell Immunity in Tumor Microenvironment. Front. Immunol..

[46] Cheng K., Cai N., Zhu J., Yang X., Liang H., Zhang W. (2022). Tumor-associated macrophages in liver cancer: From mechanisms to therapy. Cancer Commun..

[47] Han W., Lou Y., Tang J., Zhang Y., Chen Y., Li Y., Gu W., Huang J., Gui L., Tang Y. (2001). Molecular cloning and characterization of chemokine-like factor 1 (*CKLF*1), a novel human cytokine with unique structure and potential chemotactic activity. Biochem. J..

[48] Ge Y.Y., Duan H.J., Deng X.L. (2021). Possible effects of chemokine-like factor-like MARVEL transmembrane domain-containing family on antiphospholipid syndrome. Chin. Med. J..

[49] Leone P., Solimando A.G., Fasano R., Argentiero A., Malerba E., Buonavoglia A., Lupo L.G., De Re V., Silvestris N., Racanelli V. (2021). The Evolving Role of Immune Checkpoint Inhibitors in Hepatocellular Carcinoma Treatment. Vaccines.

[50] Ribas A., Wolchok J.D. (2018). Cancer immunotherapy using checkpoint blockade. Science.

[51] Qin S., Ren Z., Feng Y.H., Yau T., Wang B., Zhao H., Bai Y., Gu S., Li L., Hernandez S. (2021). Atezolizumab plus Bevacizumab versus Sorafenib in the Chinese Subpopulation with Unresectable Hepatocellular Carcinoma: Phase 3 Randomized, Open-Label IMbrave150 Study. Liver Cancer..

[52] Fu Y., Liu S., Zeng S., Shen H. (2019). From bench to bed: The tumor immune microenvironment and current immunotherapeutic strategies for hepatocellular carcinoma. J. Exp. Clin. Cancer Res..

[53] Solimando A.G., Susca N., Argentiero A., Brunetti O., Leone P., De Re V., Fasano R., Krebs M., Petracci E., Azzali I. (2022). Second-line treatments for Advanced Hepatocellular Carcinoma: A Systematic Review and Bayesian Network Meta-analysis. Clin. Exp. Med..

[54] Ruf B., Heinrich B., Greten T.F. (2021). Immunobiology and immunotherapy of HCC: Spotlight on innate and innate-like immune cells. Cell Mol. Immunol..

[55] Duffy M.J., Crown J. (2019). Biomarkers for Predicting Response to Immunotherapy with Immune Checkpoint Inhibitors in Cancer Patients. Clin. Chem..

[56] Chowell D., Yoo S.K., Valero C., Pastore A., Krishna C., Lee M., Hoen D., Shi H., Kelly D.W., Patel N. (2022). Improved prediction of immune checkpoint blockade efficacy across multiple cancer types. Nat. Biotechnol..

[57] Zhu A.X., Finn R.S., Edeline J., Cattan S., Ogasawara S., Palmer D., Verslype C., Zagonel V., Fartoux L., Vogel A. (2018). Pembrolizumab in patients with advanced hepatocellular carcinoma previously treated with sorafenib (KEYNOTE-224): A non-randomised, open-label phase 2 trial. Lancet Oncol..

