# Supplementary material for: CKLF as a Prognostic Biomarker and Its Association with Immune Infiltration in Hepatocellular Carcinoma
Source: Curr Oncol. 2023 Feb 22;30(3):2653–72. doi: 10.3390/curroncol30030202 (PMC10047849; doi:10.3390/curroncol30030202)
Supplement: Supplementary file 1 [file curroncol-30-00202-s001.zip › Supplementary Table S1.pdf]

Supplementary Table S1 Univariate and multivariate Cox proportional hazards regression analysis of CMTM family members and clinical characteristics for progression-free interval (PFI) in HCC.

Characteristics	Univariate analysis		Multivariate analysis	
	Hazard ratio (95% CI)	P value	Hazard ratio (95% CI)	P value
Age				
≤60	Reference	-	-	-
> 60	0.958 (0.706 - 1.299)	0.781	-	-
Gender				
Female	Reference	-	-	-
Male	0.943 (0.682 - 1.306)	0.725	-	-
Histologic grade				
G1	Reference	-	-	-
G2	1.054 (0.650 - 1.709)	0.832	-	-
G3	1.212 (0.739 - 1.988)	0.446	-	-
G4	1.024 (0.385 - 2.719)	0.963	-	-
Pathologic stage				
Stage I	Reference	-	Reference	-
Stage II	1.914 (1.308 - 2.801)	< **0.001**	1.878 (1.278 - 2.758)	**0.001**
Stage III	2.671 (1.852 - 3.852	< **0.001**	2.504 (1.721 - 3.644)	< **0.001**
Stage IV	5.543 (1.718 - 17.886)	**0.004**	5.874 (1.807 - 19.093)	**0.003**
CKLF	2.852 (0.673 - 12.089)	0.155	-	-
CMTM1	1.542 (1.029 - 2.309)	**0.036**	1.139 (0.694 - 1.869)	0.606
CMTM2	1.114 (0.744 - 1.667)	0.601	-	-
CMTM3	1.056 (0.916 - 1.218)	0.450	-	-
CMTM4	1.170 (1.012 - 1.353)	**0.034**	1.059 (0.898 - 1.249)	0.494
CMTM5	0.932 (0.091 - 9.532)	0.953	-	-

Characteristics	Univariate analysis		Multivariate analysis	
	Hazard ratio (95% CI)	P value	Hazard ratio (95% CI)	P value
CMTM6	1.103 (0.915 - 1.330)	0.305	-	-
CMTM7	1.210 (1.028 - 1.425)	**0.022**	1.131 (0.956 - 1.338)	0.150
CMTM8	1.149 (0.950 - 1.390)	0.152	-	-

Bold values stand for p <0.05. HR, Hazard ratio. CI, confidence interval. HCC, hepatocellular carcinoma. CMTM, Chemokine-like factor -like MARVEL transmembrane domain-containing family.
