# Supplementary material for: CKLF as a Prognostic Biomarker and Its Association with Immune Infiltration in Hepatocellular Carcinoma
Source: Curr Oncol. 2023 Feb 22;30(3):2653–72. doi: 10.3390/curroncol30030202 (PMC10047849; doi:10.3390/curroncol30030202)
Supplement: Supplementary file 1 [file curroncol-30-00202-s001.zip › Supplementary Table S2.pdf]

Supplementary Table S2 The relationship between CKLF expression and clinicopathological characteristics in HCC patients

Characteristics	*No. of patients*	CKLF Expression		*p*-value
		Low n=15	High n=26	
Age（years）				0.309
＜60	15	7	8	
≥60	26	8	18	
Gender				0.475
Male	30	10	20	
Female	11	5	6	
HBsAg				0.664
Negative	12	5	7	
Positive	29	10	19	
Child-Pugh classification				0.3
A	23	10	13	
B	18	5	13	
AFP				0.548
≤ 400ng/ml	26	9	18	
＞ 400ng/ml	15	6	8	
Liver cirrhosis				0.475
Absent	11	5	6	
Present	30	10	20	
Tumor number				0.485
Single	28	9	19	
Multiple	13	6	7	
TNM stage				**0.024**
I/II	28	7	21	
III/IV	13	8	5	

Bold values are statistically significant, $p < 0.05$. HCC, hepatocellular carcinoma.
