# Supplementary material for: CKLF as a Prognostic Biomarker and Its Association with Immune Infiltration in Hepatocellular Carcinoma
Source: Curr Oncol. 2023 Feb 22;30(3):2653–72. doi: 10.3390/curroncol30030202 (PMC10047849; doi:10.3390/curroncol30030202)
Supplement: Supplementary file 1 [file curroncol-30-00202-s001.zip › Supplementary Table S3.pdf]

Supplementary Table S3 Univariate and multivariate analyses of overall survival in HCC patients (Cox proportional hazards regression model)

Characteristics	Univariate analysis		Multivariate analysis	
	Hazard ratio (95% CI)	P value	Hazard ratio (95% CI)	P value
Age				
> 60	Reference	-	-	-
<= 60	0.649 (0.275-1.532)	0.324	-	-
Gender				
Male	Reference	-	-	-
Female	0.622 (0.496-3.236)	0.622	-	-
HBsAg				
Positive	Reference	-	-	-
Negative	0.521 (0.216-1.258)	0.147	-	-
Child-Pugh classification				
B	Reference	-	-	-
A	0.524(0.225-1.221)	0.134	-	-
AFP				
≤ 400ng/ml	Reference	-	-	-
> 400ng/ml	2.362 (0.939-5.942)	0.068	-	-
Liver cirrhosis				
Absent	Reference	-	-	-
Present	2.566 (0.942-6.990)	0.065	-	-
Tumor number				
Multiple	Reference	-	-	-
Single	0.943 (0.413-2.151)	0.889	-	-
Tumor number				

Characteristics	Univariate analysis		Multivariate analysis	
	Hazard ratio (95% CI)	P value	Hazard ratio (95% CI)	P value
Multiple	Reference	-	-	-
Single	0.943 (0.413-2.151)	0.889	-	-
TNM stage				
I/II	Reference	-	Reference	-
III/IV	2.604(1.106-6.131)	**0.028**	2.908(1.110-7.616)	**0.030**
CKLF expression				
Low	Reference	-	Reference	-
High	3.503(1.417-8.658)	**0.007**	5.434(1.946-15.171)	**0.001**

Bold values stand for p <0.05. HR, Hazard ratio. CI, confidence interval. HCC, hepatocellular carcinoma.
