# Supplementary figures and images for: CKLF as a Prognostic Biomarker and Its Association with Immune Infiltration in Hepatocellular Carcinoma
Source: Curr Oncol. 2023 Feb 22;30(3):2653–72. doi: 10.3390/curroncol30030202 (PMC10047849; doi:10.3390/curroncol30030202)

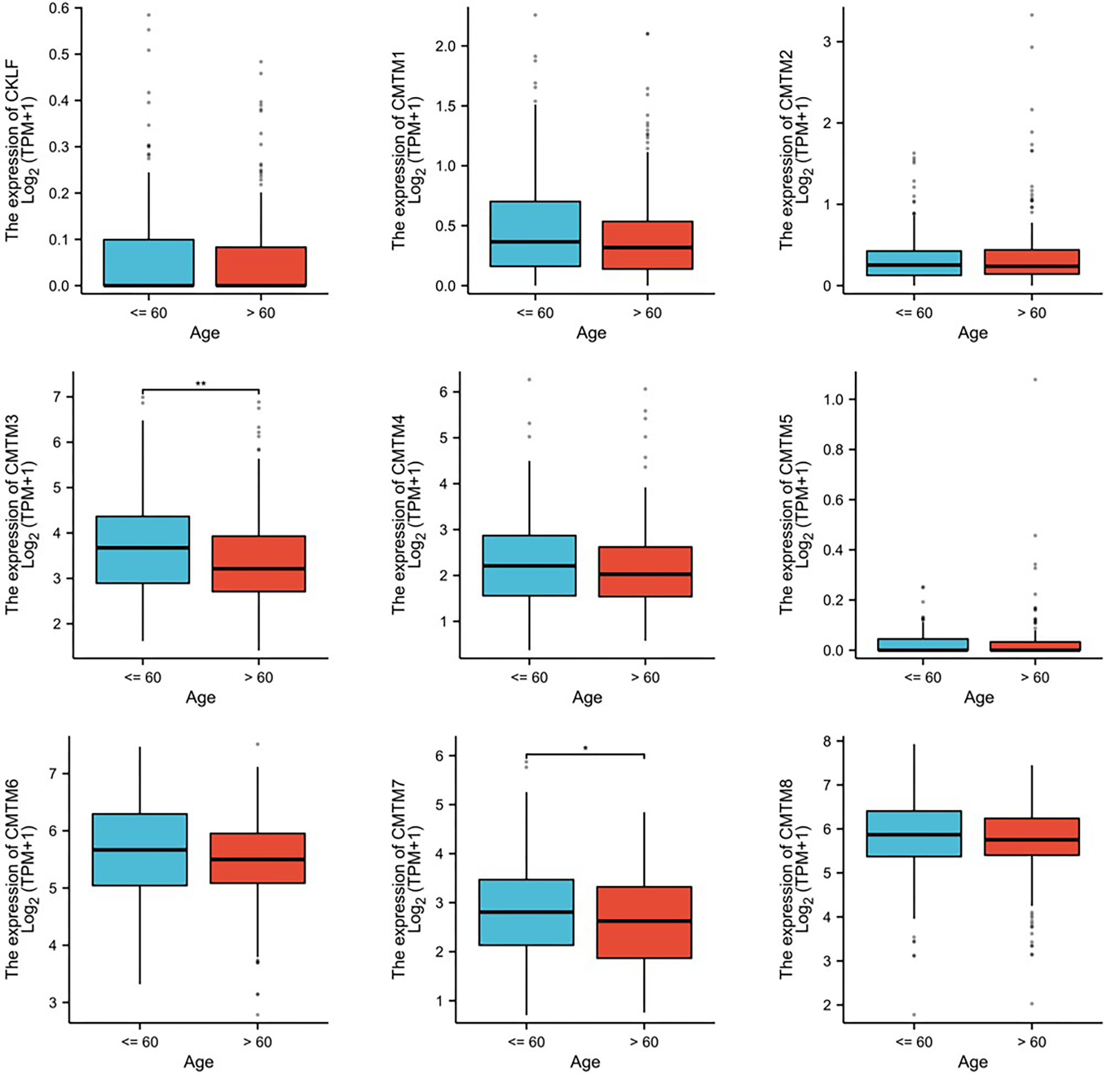

Supplement: Supplementary file 1 [file curroncol-30-00202-s001.zip › Supplementary Figure S1.tif]

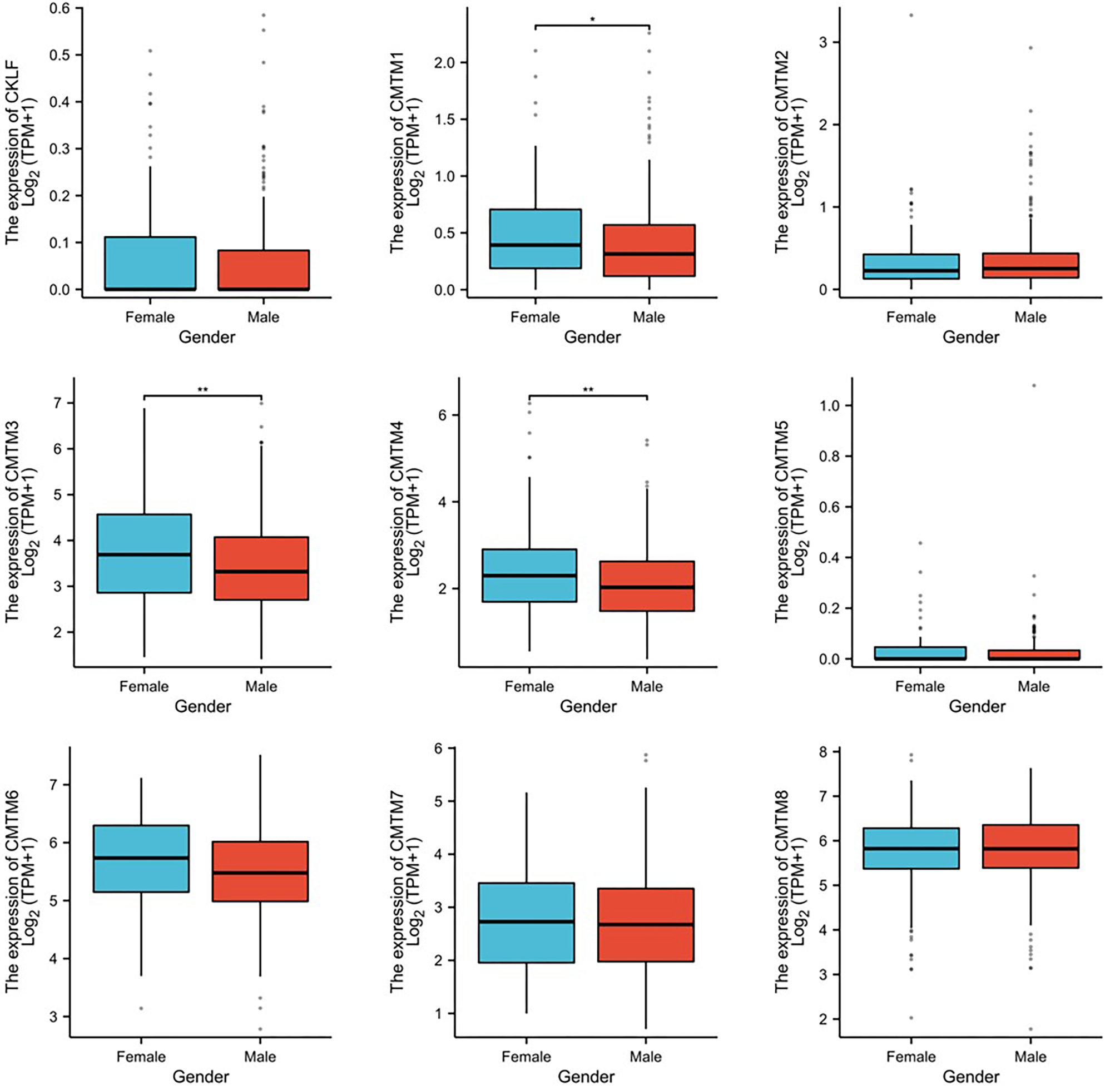

Supplement: Supplementary file 1 [file curroncol-30-00202-s001.zip › Supplementary Figure S2.tif]

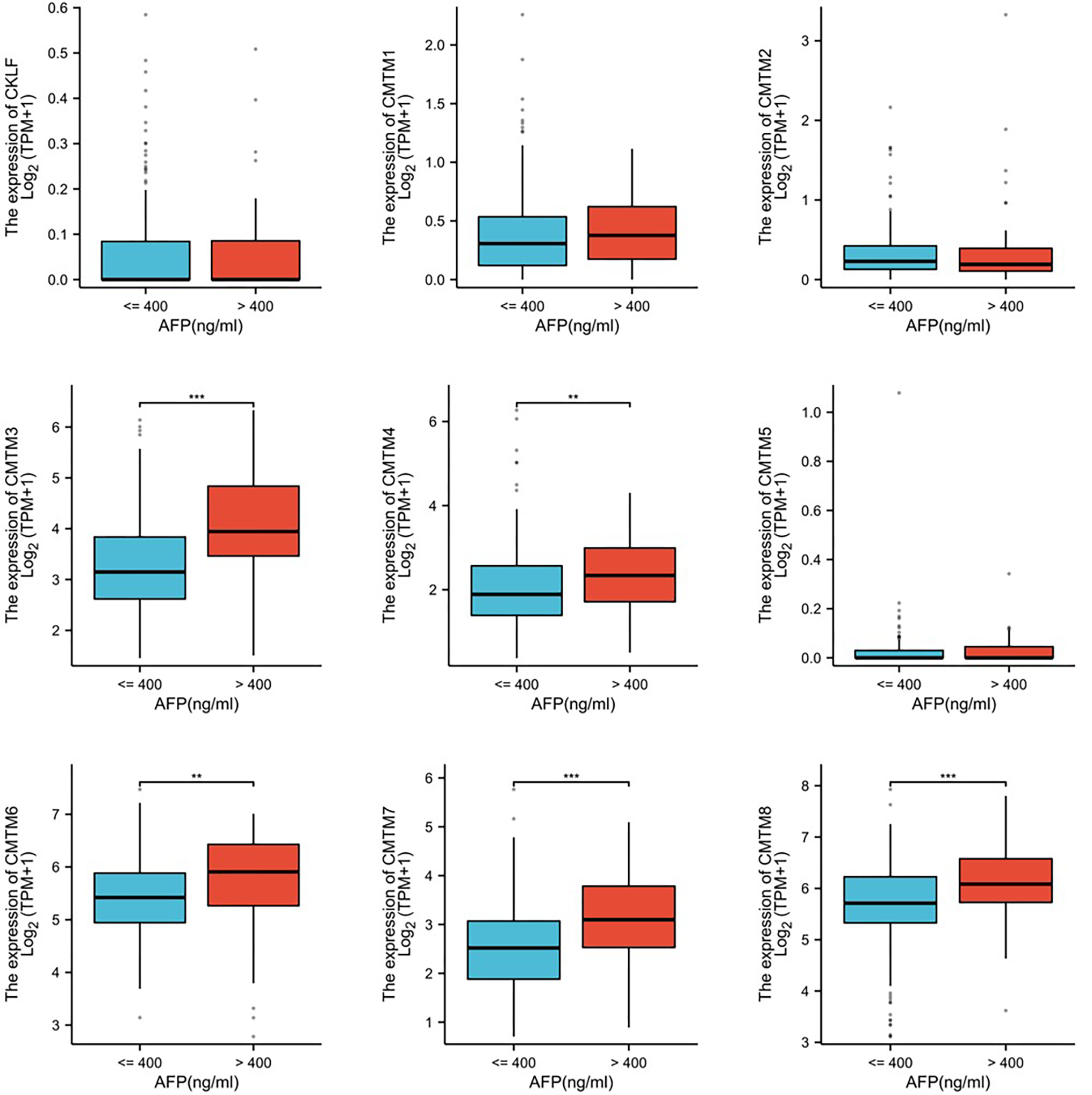

Supplement: Supplementary file 1 [file curroncol-30-00202-s001.zip › Supplementary Figure S3.tif]

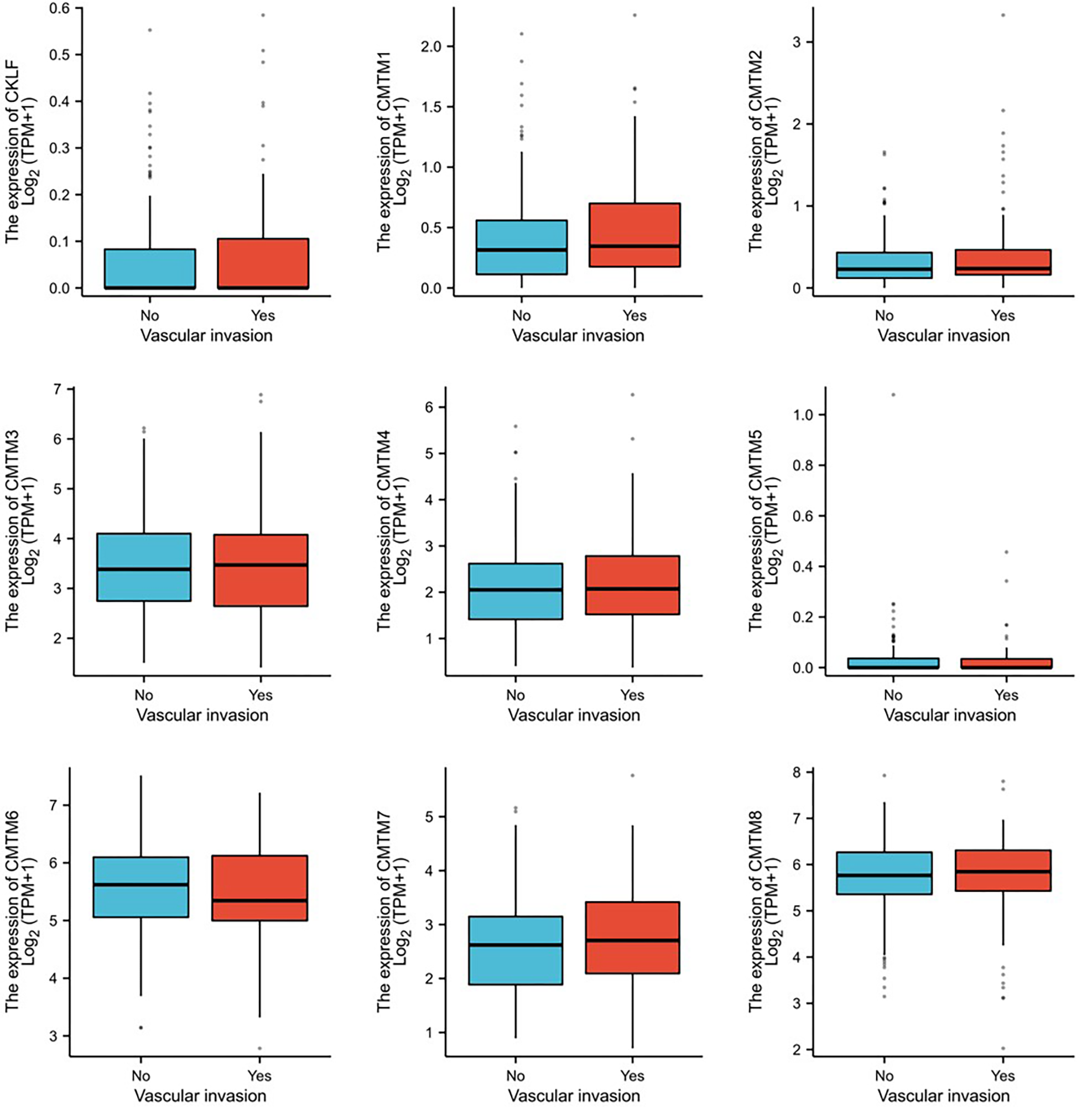

Supplement: Supplementary file 1 [file curroncol-30-00202-s001.zip › Supplementary Figure S4.tif]

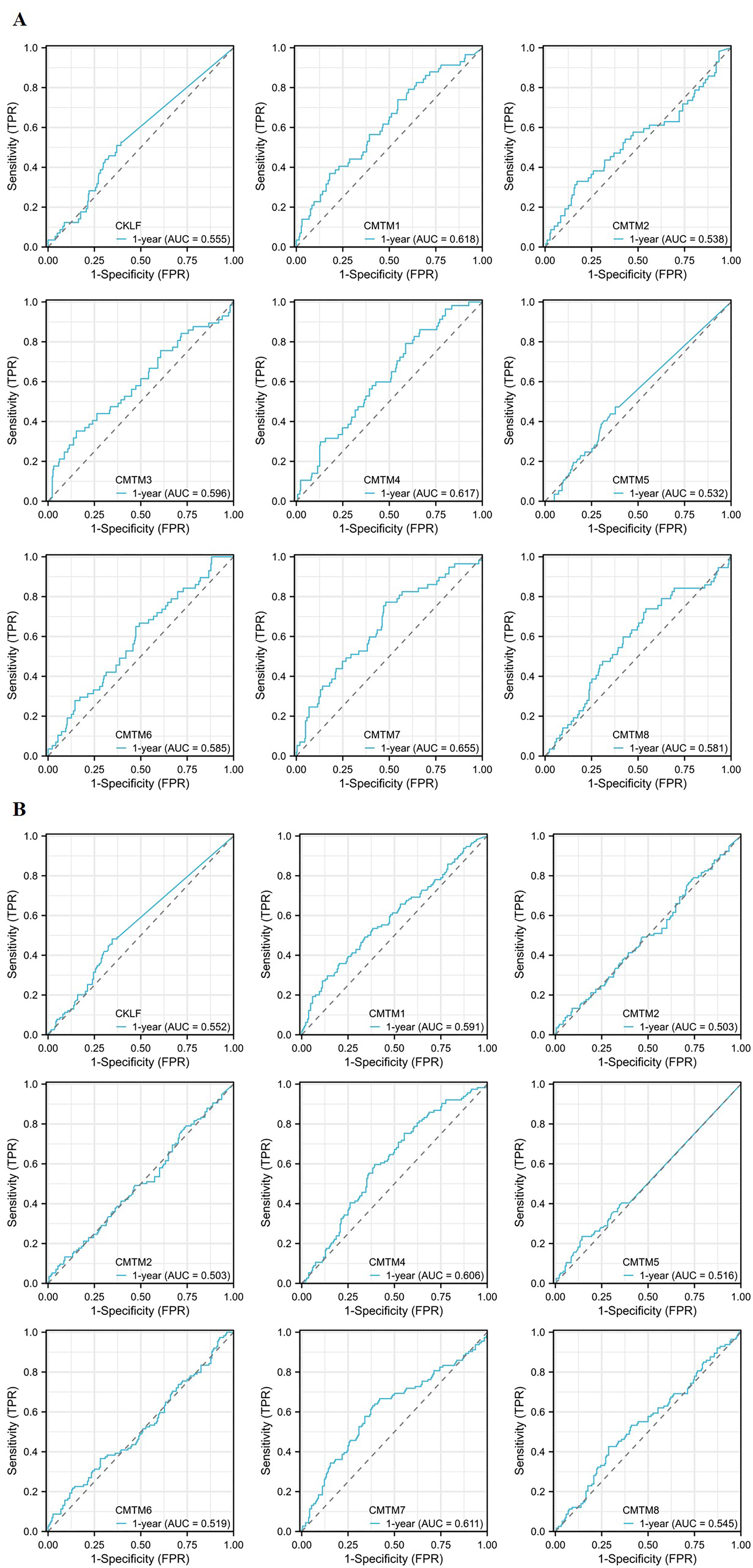

Supplement: Supplementary file 1 [file curroncol-30-00202-s001.zip › Supplementary Figure S5.tif]

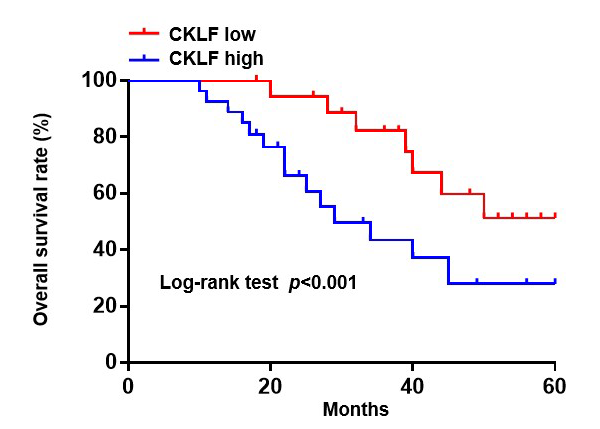

Supplement: Supplementary file 1 [file curroncol-30-00202-s001.zip › Supplementary Figure S6.tif]
